# Chemistry, Biosynthesis and Pharmacology of Sarsasapogenin: A Potential Natural Steroid Molecule for New Drug Design, Development and Therapy

**DOI:** 10.3390/molecules27062032

**Published:** 2022-03-21

**Authors:** Nur Hanisah Mustafa, Mahendran Sekar, Shivkanya Fuloria, M. Yasmin Begum, Siew Hua Gan, Nur Najihah Izzati Mat Rani, Subban Ravi, Kumarappan Chidambaram, Vetriselvan Subramaniyan, Kathiresan V. Sathasivam, Srikanth Jeyabalan, Subasini Uthirapathy, Sivasankaran Ponnusankar, Pei Teng Lum, Vijay Bhalla, Neeraj Kumar Fuloria

**Affiliations:** 1 Department of Pharmaceutical Chemistry, Faculty of Pharmacy and Health Sciences, Royal College of Medicine Perak, Universiti Kuala Lumpur, Ipoh 30450, Malaysia; nhanisah.mustafa@s.unikl.edu.my (N.H.M.); peiteng1013@gmail.com (P.T.L.); 2 Faculty of Pharmacy, AIMST University, Bedong 08100, Malaysia; 3 Department of Pharmaceutics, College of Pharmacy, King Khalid University, Abha 61421, Saudi Arabia; ybajen@kku.edu.sa; 4 School of Pharmacy, Monash University Malaysia, Bandar Sunway, Kuala Lumpur 47500, Malaysia; gan.siewhua@monash.edu; 5 Faculty of Pharmacy and Health Sciences, Royal College of Medicine Perak, Universiti Kuala Lumpur, Ipoh 30450, Malaysia; najihah.izzti@gmail.com; 6 Department of Chemistry, Karpagam Academy of Higher Education, Coimbatore 641021, Tamil Nadu, India; ravisubban@rediffmail.com; 7 Department of Pharmacology, College of Pharmacy, King Khalid University, Abha 62529, Saudi Arabia; kumarappan@kku.edu.sa; 8 Faculty of Medicine, Bioscience and Nursing, MAHSA University, Jalan SP 2, Bandar Saujana Putra, Jenjarom 42610, Malaysia; drvetriselvan@mahsa.edu.my; 9 Faculty of Applied Sciences, AIMST University, Bedong, Sungai Petani 08100, Malaysia; skathir@aimst.edu.my; 10 Department of Pharmacology, Sri Ramachandra Faculty of Pharmacy, Sri Ramachandra Institute of Higher Education and Research (DU), Porur, Chennai 600116, Tamil Nadu, India; srikanth.j@sriramachandra.edu.in; 11 Faculty of Pharmacy, Tishk International University, Erbil 44001, Kurdistan Region, Iraq; subasini.uthirapathy@tiu.edu.iq; 12 Department of Pharmacy Practice, JSS College of Pharmacy, JSS Academy of Higher Education & Research, Ooty 643001, Tamil Nadu, India; ponnusankarsivas@gmail.com; 13 SGT College of Pharmacy, SGT University, Gurugram 122505, Haryana, India; vijay_0434@hotmail.com; 14 Center for Transdisciplinary Research, Department of Pharmacology, Saveetha Institute of Medical and Technical Sciences, Saveetha Dental College and Hospital, Saveetha University, Chennai 600077, Tamil Nadu, India

**Keywords:** sarsasapogenin, steroid, phytochemistry, biosynthesis, pharmacology, drug development

## Abstract

Sarsasapogenin is a natural steroidal sapogenin molecule obtained mainly from *Anemarrhena asphodeloides* Bunge. Among the various phytosteroids present, sarsasapogenin has emerged as a promising molecule due to the fact of its diverse pharmacological activities. In this review, the chemistry, biosynthesis and pharmacological potentials of sarsasapogenin are summarised. Between 1996 and the present, the relevant literature regarding sarsasapogenin was obtained from scientific databases including PubMed, ScienceDirect, Scopus, and Google Scholar. Overall, sarsasapogenin is a potent molecule with anti-inflammatory, anticancer, antidiabetic, anti-osteoclastogenic and neuroprotective activities. It is also a potential molecule in the treatment for precocious puberty. This review also discusses the metabolism, pharmacokinetics and possible structural modifications as well as obstacles and opportunities for sarsasapogenin to become a drug molecule in the near future. More comprehensive preclinical studies, clinical trials, drug delivery, formulations of effective doses in pharmacokinetics studies, evaluation of adverse effects and potential synergistic effects with other drugs need to be thoroughly investigated to make sarsasapogenin a potential molecule for future drug development.

## 1. Introduction

Since ancient times, natural products have been a key source of pharmaceuticals, and many of them have been approved as effective drugs or drug candidates. Plants, animals, marines and microbes are the most common sources of natural products. Plants are living chemical factories, synthesizing an enormous variety of secondary metabolites that can serve as the basis for numerous commercial pharmaceutical preparations. Although the various chemical elements of medicinal plants have biological activities that are beneficial to health via the pharmaceutical and food industries, they also have significant values in the perfume, agrochemical and cosmetic businesses [1,2,3]. Drug discovery from natural origin entails a number of steps including botanical identification and collection, extraction, isolation, purification, and structure elucidation of the desired bioactive molecule as well as its biological evaluation. Formulation development, stability testing of the established formulation, preclinical and clinical trial investigations and the submission of a New Drug Application (NDA) are all steps in the process [4,5].

*Anemarrhena asphodeloides* Bunge, commonly referred to as Zhimu (Rhizoma *Anemarrhenae*), is a member of the *Anemarrhena* genus in the Liliaceae family [6] and is found primarily in China, Japan, Korea and other eastern Asian nations [7,8,9]. The rhizomes have been used to cure febrile diseases, hyperpyrexia, fever, cough, polydipsia, osteopyrexia and constipation since ancient times [10]. *Anemarrhena asphodeloides* is predominantly composed of steroidal saponins including sarsasapogenin [11]. Sarsasapogenin has been reported for many pharmacological actions and widely investigated for anti-inflammatory, neuroprotective and memory impairment related to ageing [12,13,14,15]. Additionally, its modified structure and a number of derivatives identified have been confirmed to have biological properties such as antitumour activity, antidepressant activity, anxiolytic-like action and an Aβ-reducing effect [16,17,18,19,20,21]. Despite its variable therapeutic properties, there has been no thorough and comprehensive review on sarsasapogenin. Hence, the phytochemistry, biosynthesis and physicochemical characteristics of sarsasapogenin are summarised in this review with a focus on its biological activities. This review discusses the metabolism, pharmacokinetics and potential structural modifications, as well as the limitations and prospects for sarsasapogenin becoming a drug molecule. The scientific evidence is anticipated to provide a solid foundation for future research as well as vital information for the drug development of sarsasapogenin as a therapeutic agent and health product.

## 2. Phytochemistry of Sarsasapogenin

Sarsasapogenin is present in many medicinal plants and the details are summarised in Table 1.

## 3. Isolation of Sarsasapogenin

The following is the simplest approach for isolating sarsasapogenin from *Anemarrhenae asphodeloides* among the several methods available. The dry powder form of *Anemarrhenae asphodeloides* rhizome (1 kg) was extracted for 4 h at 70 °C with 20 L of 95% aqueous ethanol. The ethanolic extract was then concentrated using a rotary evaporator at a specific temperature and pressure. After, the extract was then packed in a column chromatography on macroporous resin and eluted with 10%, 30%, 50%, and 90% ethanol. The 90% ethanol fractions were concentrated using a rotary evaporator at a controlled temperature and pressure. After mixing the concentrated solution with an equal quantity of 10% HCl, it was incubated for 2 h at 50 °C. After concentration, the residue should be dissolved in absolute ethanol and decolorised for 30 min with activated carbon. The crystalline form of sarsasapogenin was then filtrated, saturated with absolute ethanol, and kept at room temperature. The formation of white acicular crystals of sarsasapogenin (4.6 g, 0.46%) was obtained after repeating the recrystallisation technique as mentioned by Bao et al. [29].

## 4. Chemistry of Sarsasapogenin

**Figure 1:**
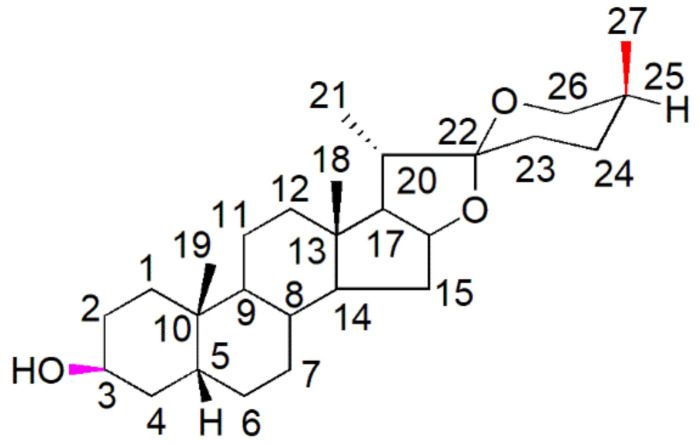
Chemical structure of sarsasapogenin. Each carbon atom’s numbering system is displayed in the structure. A hydroxyl group is attached at the C3 position (in purple). At the other end of the rings C-25, a chiral atom is bound to a methyl group (C-27) in the S-configuration (in red).

Spirostane saponins are monodesmosidic glycosides with six rings (A–F) that are similar to sapogenin but differ in having an axially oriented methyl or hydroxymethyl (C-27) on the F-ring. Sarsasapogenin is a spirostane glycoside sapogenin that has a 5β configuration between rings A and B (Figure 1).

Based on electron spray ionisation mass spectrometry (ESI-MS), sarsasapogenin produced a molecular ion peak at *m*/*z* 417.34 [M + H]^+^ ion corresponding to a molecular formula of C_27_H_44_O_3_. The infrared (IR) spectra exhibited characteristic absorption bands at 3407 (–OH), 2931, 1449, 1378 (–C–H) and at 1069 cm^−1^, characteristic of C–O groups. In the proton nuclear magnetic resonance (H-NMR) spectra, the H-3 proton appeared at δ 4.10 as a broad singlet. The doublet of doublet signals appeared at δ 3.94 (dd, *J* = 11.0, 2.8 Hz) and 3.29 (d, *J* = 10.9 Hz) each integrating for one proton, attributed to H-26 methylene protons. The two singlets appeared at δ 0.75 and 0.97 each integrating for three protons due to the H-18 and H-19 methyl groups, respectively. The pair of doublets at 0.98 (3H, d, *J* = 6.2 Hz) and 1.07 (3H, d, *J* = 7.4 Hz) were assigned to the H-21 and H-27 methyl groups respectively. The triplet of doublets signals at δ 4.40 (1H, td, *J* = 7.9, 7.3, 6.2 Hz) were attributed to H-16 [45,46]. All other signals closely resemble a steroidal nucleus. In the ^13^C-NMR spectra, the methyl carbons C-18, C-19, C-21 and C-27 appeared at δ 16.2, 24.1, 14.5 and at 16.6, respectively. The carbon under oxygen function C-3, C-16, C-22 and C-26 resonated at δ 67.2, 81.2, 109.9 and at 65.3, respectively. The rest of the signals were assigned as in Table 2. The above assignments were made based on distortion-less enhancement by polarisation transfer (DEPT), heteronuclear multiple quantum coherence (HMQC), and heteronuclear multiple bond coherence (HMBC) experiments. Four methyl carbons, eleven methylene carbons, nine methine carbons and three quaternary carbons were found in the DEPT experiment.

## 5. Biosynthesis of Sarsasapogenin

**Figure 2:**
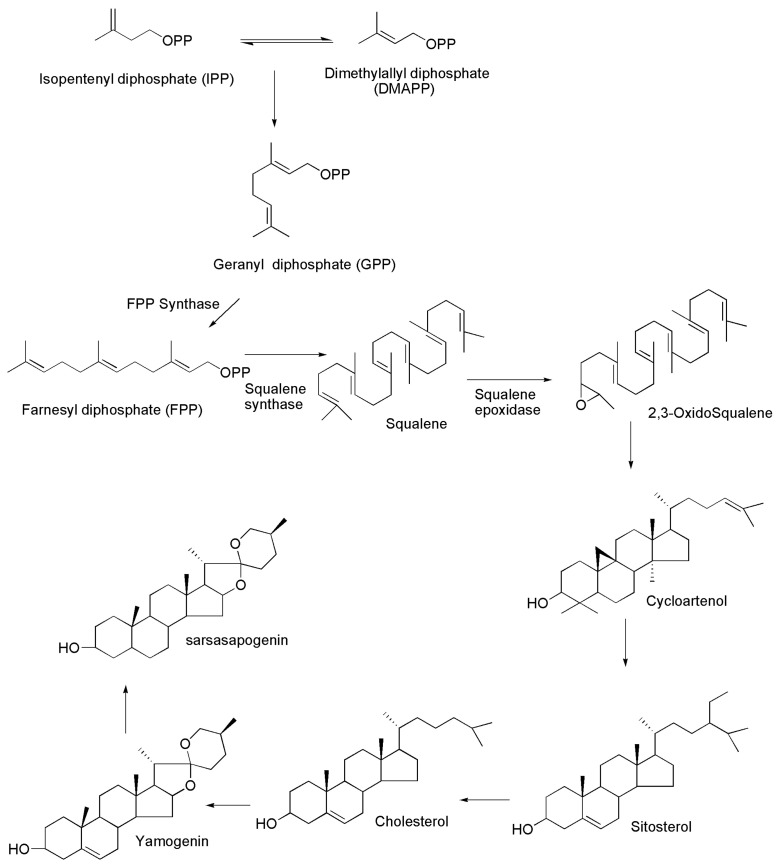
Biosynthesis of sarsasapogenin.

The biosynthetic mechanism, chemical structure and therapeutic actions of steroidal saponins remain elusive despite countless investigations. Glycosylation is assumed to be the final step in the production of steroidal saponins and to have a role in regulating the biological activity. Nevertheless, the isolation of glycosyltransferases that catalyse the transfer of sugar molecules to steroidal compounds will aid in the understanding of the mechanisms that produce and regulate saponin activity in plants. In plants, sapogenins occur in the form of their glycosides, i.e., the saponins. Acid saponins have triterpenoid structures, whereas neutral saponins are steroid derivatives with spiroketal side chains; both have similar biogenesis process. However, following the production of the triterpenoid hydrocarbon–squalene, a branch appears, leading to cyclic triterpenoids in one direction and spiroketal steroids in the other. Figure 2 depicts the biosynthesis of squalene, cholesterol and different steroidal molecules including aglycones.

Isopentenyl diphosphate (IPP) and dimethylallyl diphosphate (DMAPP), formed by the mevalonate (MVA) or methylerythritol pathways (MEPs), are used to produce sarsasapogenin [47,48,49,50]. DMAPP is converted to geranyl diphosphate (GPP) by geranyl diphosphate synthase and, subsequently, to farnesyl diphosphate (FPP) by farnesyl diphosphate synthase (FDS) [51,52]. The main branching point in the biosynthesis of sarsasapogenins is farnesyl diphosphate (FPP). Squalene synthase catalyses the head-to-head condensation of two units of farnesyl diphosphate to create a 30-carbon linear squalene. Squalene is then transformed to squalene epoxide by squalene epoxidase. Subsequently, the latter is cyclised by a variety of triterpene cyclases by protonation and an epoxide ring opening by a number of triterpene cyclases. With the help of cycloartenol synthase, tetracyclic cycloartenol is formed, serving as a precursor for the biosynthesis of steroidal saponin. Moreover, cycloartenol undergoes a series of rearrangements to form sitosterol, which are catalysed by enzymes such as cycloeucalenol cycloisomerase, methylsterol monooxygenase, sterol 14 alpha-demethylase, 7-dehydrocholesterol reductases and lanosterol oxidase, among others. Sitosterol is a direct precursor for the manufacture of steroidal saponins and is glucosylated at different locations to create saponins, which are catalysed by glucosyltransferases [53,54,55].

## 6. Physicochemical and Drug-Likeness Properties of Sarsasapogenin

For drug molecules to enter their active sites, several obstacles need to be overcome. Complex biological processes can be presented using physiochemical characteristics, and knowing them can help to move a drug development programme forward in both the lead optimisation and lead identification phases. The physicochemical properties of sarsasapogenin were primarily derived from PubChem and other reliable databases. Biovia Discovery studio 19.0 was used to determine the drug-likeness parameters (molecular weight, H-bond donors, H-bond acceptors, log *P*-value and rotatable bonds) as indicated by Lipinski’s rule of five (Table 3).

## 7. Biological Properties of Sarsasapogenin

### 7.1. Anti-Inflammatory Activity

**Figure 3:**
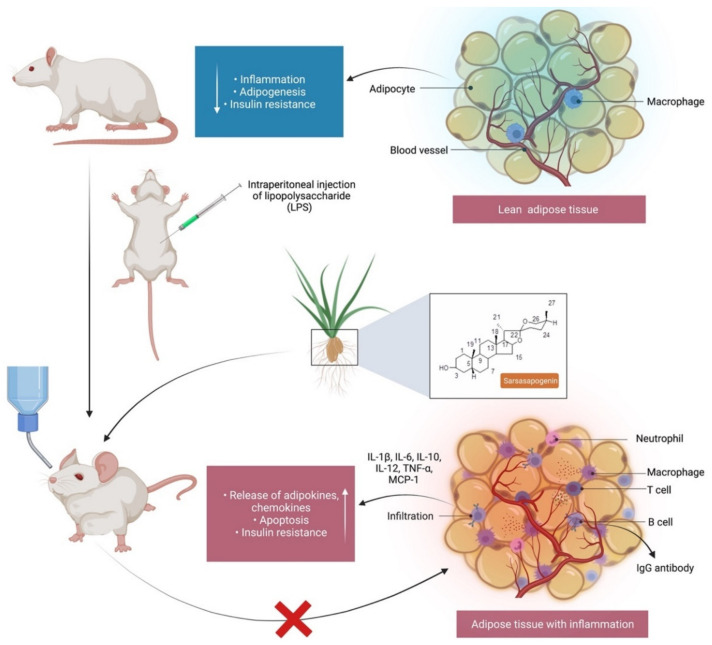
Sarsasapogenin’s anti-inflammatory action in adipose tissue. While lean adipose tissue contributes to metabolic balance, inflammation regulation and insulin resistance, as obesity progresses, adipocytes enlarge and release adipokines, resulting in an increase in the production of pro-inflammatory factors and immune cell infiltration. Sarsasapogenin has been shown to inhibit pro-inflammatory cytokines, such as TNF-α, IL–1β, IL–6, IL-10, IL-12 and MCP-1, in white adipose tissue. TNF-α, tumour necrosis factor alpha; interleukin-1 beta, -6, -10 and -12; MCP-1, monocyte chemoattractant protein-1.

**Figure 4:**
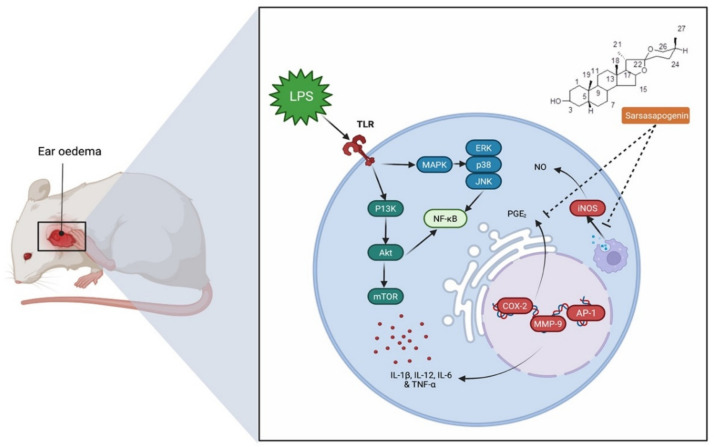
Mechanism of inhibition exhibited by sarsasapogenin in ear oedema. Sarsasapogenin reduced iNOS expression and PGE2 levels, which suppressed the inflammation generated by LPS and ameliorated ear oedema. MAPK, mitogen-activated protein kinase; ERK, extracellular-signal-regulated kinase; JNK, c-Jun N-terminal kinase; NF-kB, nuclear factor kappa-light-chain-enhancer of activated B cell; P13K, phosphatidylinositol-3-kinase; Akt, Ak strain transforming; mTOR, mammalian target of rapamycin; TLR, Toll-like receptor.

Inflammation is an essential protective process that protects organisms from physical, chemical and infectious risks. However, it is common for the inflammatory response to many exposures to erroneously damage normal tissues [56]. Nonsteroidal anti-inflammatory drugs (NSAIDs) are commonly used to treat inflammation, have a side effect profile, despite their significant function in pain and inflammation management [57]. This highlights the need for novel anti-inflammatory drugs that are both safety and efficacy. Yu et al. [58] confirmed the anti-inflammatory activity of sarsasapogenin that enters adipose tissue following a single oral administration to inhibit acute adipose inflammation caused by an intraperitoneal injection of lipopolysaccharide (LPS) to C57BL/6J mice (Figure 3). LPS contains bacterial endotoxin broadly used for observing the response of acute inflammation in mice, since it stimulates the production of cytokines such as tumour necrosis factor alpha (TNF-α) and interleukin-1 (IL)-1β and IL-6. Pre-treatment with sarsasapogenin (80 mg/kg) for 18 days ameliorates TNF-α, IL-1β and IL-6 levels in the plasma and reduces the concentration of the anti-inflammatory cytokine-10. Other than releasing cytokines, LPS binds to Toll-like receptors to stimulate the IKK/nuclear factor kappa B (NF-κB) and the JNK signalling pathways and elevate both the expression and secretion of pro-inflammatory genes. Sarsasapogenin can supress the expression of the pro-inflammatory genes, including TNF-α, IL–1β and IL-6, monocyte chemo-attractant protein-1 (MCP-1), nitric oxide synthase 2 (Nos2) and cyclooxygenase-2 (COX2), in white adipose tissue, simultaneously increasing the transcription of M2, including Arg1, YM1, Fizz1 and IL-10, which further stimulate anti-inflammatory cytokines. Moreover, sarsasapogenin reduces NF-κB, its inhibitor (IKK) and c-Jun N terminal kinase (JNK) phosphorylation, which contribute to inflammation in addition to increasing the protein level of nuclear factor of kappa light polypeptide gene enhancer in B-cell inhibitor, alpha (IκB-α), which allows inhibition of NF-κB transcription factor. Overall, the findings indicate that sarsasapogenin can cause inhibition of acute inflammatory response induced by LPS, ameliorate the inflammatory status of adipose tissue, and halt the inflammatory signalling pathways of IKK/NF-κB and JNK when taken orally [58]. Further, Dong et al. [12] reported that sarsasapogenin inhibits the inflammation induced by LPS and relieves ear oedema by (1) reducing the production of nitric oxide (NO) through reduction of nitric oxide synthase (iNOS) expression in macrophages and (2) reducing prostaglandin E2 (PGE_2_) levels induced by LPS (Figure 4). Nitrogen dioxide (NO_2_) plays an important role in the inflammatory process. NO is generated by nitric oxide synthases (NOS), while iNOS is expressed in macrophages and is activated by the presence of pro-inflammatory cytokines. PGE_2_ is a crucial component of arachidonic acid metabolism catalysed by COX-2. Dong et al. [12] reported that although LPS stimulation increases PGE_2_ levels, sarsasapogenin inhibits its production in a dose-dependent manner, overall indicating that the suppression of NO and PGE_2_ production by sarsasapogenin are contributed by the inhibition of iNOS and COX. Although TNF-α is also responsible for increasing the levels of COX-2 and iNOS, sarsasapogenin inhibits the level of TNF-α to reduce COX-2 and iNOS levels.

Lim et al. [28] conducted a study on timosaponin AIII and its metabolite sarsasapogenin in improving colitis in rats by (1) suppressing NF-κB and mitogen-activated protein kinase (MAPK) activation as well as (2) restoring T-helper 17/regulatory T (Th17/Treg) cell balance. Stimulation of LPS or peptidoglycan induces inflammation by activating the canonical and non-canonical Toll-like receptor (TLR)-NF-κB signalling pathways. The activation starts a signalling cascade via the Toll/IL-1R (TIR) that leads to the activation of interleukin-1R-associated kinases (IRAKs). Phosphorylated IRAK1 activates a multimeric protein complex, TNF receptor-associated Factor 6 (TRAF6), transforming growth factor-β-activated kinase 1 (TAK1), TAK1-binding protein 1 (TAB1), TAK1-binding protein 2 (TAB2), triggering NF-κB activation as well as the production of TNF-α and IL-1β. Although LPS stimulated the macrophages, both AIII and sarsasapogenin suppressed NF-κB and MAPK activation as well as IRAK1, TAK1, and IκBα phosphorylation. It was concluded that modulating the NF-κB signalling pathway may be advantageous in treating chronic inflammation. AIII and sarsasapogenin also reduced LPS binding to macrophage Toll-like receptor 4 as well as the polarisations of M2 to M1 macrophages. Oral administration of AIII and sarsasapogenin attenuated TNBS-induced colon shortening and myeloperoxidase activity in mice as well as decreased NF-κB activation and (IL)-1β, TNF- α, and IL-6 levels while simultaneously raising IL-10. In colonic tissue, both drugs suppressed Th17 cell development. Additionally, AIII and sarsasapogenin reduced the in vitro development of splenic CD4 30+ T cells into Th17 cells. Comparatively, sarsasapogenin had stronger anti-inflammatory effects both in vitro and in vivo compared to AIII. Overall, the findings imply that orally administered AIII may be converted to sarsasapogenin by the gut microbiota to alleviate inflammatory diseases, such as colitis, by blocking the TLR4-NF-κB/MAPK signalling pathway while restoring the Th17/Treg cell balance [28]. In another study, Mandlik et al. [40] investigated chronic inflammatory bowel diseases, such as ulceritis colitis (UC), caused by oxido-nitrosative stress and the release of pro-inflammatory cytokines that affect the colon’s mucosal lining. Administration of sarsasapogenin (50 µg) to rats with 2,4,6-trinitrobenzene sulfonic acid (TNBS)-induced UC remarkably reduced the colon weight/length ratio, macroscopic inflammation score, lesions score, diarrhoea score, and adhesion scores. Moreover, biochemical parameters, such as the pro-inflammatory cytokines, haematological parameters, serum IgE levels, and oxidative stress markers were all successfully reduced following sarsasapogenin treatment. There was also significant improvement in the histopathological findings following sarsasapogenin treatment.

Overall, sarsasapogenin’s anti-inflammatory activity appears to be mainly due to the reduction in oxidative stress and inhibition of TNF-α release. Overall, the findings imply that sarsasapogenin could be a promising drug-like treatment for a wide range of inflammatory disorders.

### 7.2. Anticancer Activity

Bao et al. [29] proposed that sarsasapogenin can be used as an anti-liver cancer drug-like molecule due to the fact of its antitumour effect. They reported that sarsasapogenin (1 mg/mL) has remarkable toxicity against HepG2 human hepatoma cells, where cell viability has shown a 50% minimum inhibitory concentration (IC_50_) of 42.4 µg/mL for 48 h by distinct dose- and time-dependent diminution. There was apoptosis to HepG2 cells as characterised by the escalation of the G2/M period cell, chromatin condensation, nuclear indentations and withdrawal of membrane microvilli, cytoplasmic mass declination and generation of apoptosis bodies as well as shrinkage of cells as seen under the electron microscope. Based on the study, sarsasapogenin suppresses tumour growth by inducing HepG2 cell apoptosis along with G2/M arrest. Thus, sarsasapogenin may be a potential treatment against cancer due to the dual mechanism, in addition to the fact that it is a highly safe compound [29]. Moreover, Ni et al. [59] indicated that mitochondrial relative oxygen stress (ROS) burst is an early sign in sarsasapogenin-induced apoptosis in HepG2 cells, where prolonged generation of ROS causes dysfunction of mitochondria and cytochrome c (cyt c). In fact, there was an almost instantaneous burst of ROS within 20 s following sarsasapogenin (25 mg/mL) administration, for a further 8 min. Based on the findings of the redox study, the prolonged exposure of ROS was followed by a decrease in the intracellular glutathione (GSH) level for 2 h. Furthermore, Bai and Cederbaum [60] stated that GSH reduction occurs as a consequence of ROS generation, hence, causing mitochondrial dysfunction [61]. The increase in ROS coupled with mitochondria dysfunction triggers the release of cyt c [62], which is an essential component of the mitochondria electron transfer chain that turn on the downstream of apoptotic executioner and caspase. This fact confirms that cyt c is important in apoptotic events occurring by ROS burst, further causing mitochondria dysfunction and the release of cyt c. Overall, sarsasapogenin induced early apoptosis of tumour HepG2 cell by (1) bursting of ROS; (2) prolonged generation of ROS; (3) mitochondria dysfunction; (4) release of cyt c.

Sarsasapogenin induced apoptosis through an ROS-mediated mitochondrial pathway and endoplasmic reticulum (ER) stress pathway in HeLa cells. ER is responsible in apoptosis regulation where ER stress can stimulate several signalling pathways including ER-associated protein degradation and unfolded protein response (URP). After HeLa cells were cultured with or without sarsasapogenin (60 µM), the stress sensor of ER, such as protein kinase RNA-like ER kinase (PERK) and elF21, were phosphorylated at an early stage within 6 h of treatment with sarsasapogenin. However, the level of PERK and eukaryotic translation initiation factor 2 subunit 1 (elF21) remained unchanged. Additionally, the activating transcription factor 6 (ATF6), which is also a protein of ER, was cleaved and turned into the activated form or ATF6 fragmentation. Additionally, ER chaperon, such as GR78 and GRP94, were also stimulated. The transduction signalling pathways were upregulated to counteract the burden of ER by impeding protein synthesis. Nevertheless, should the ER stress continue for a longer period and become even more severe, these conditions can induce apoptosis [63]. Sarsasapogenin can also cause C/EBP homologous protein (CHOP), a key transcription factor stimulated by ER to be activated as well as the overexpression of CHOP, resulting in growth arrest and apoptosis. Overall, the findings indicate that sarsasapogenin induces apoptosis by an ER stress. Apart from that, ROS, which are mediators of intracellular cascades, can disintegrate the mitochondrial membrane, leading to apoptosis. Consequently, the overproduction of ROS, leads to oxidative stress, cell dysfunction, necrosis and apoptosis. All of the above, indicates that sarsasapogenin induced apoptosis by ER stress, ROS formation and mitochondria dysfunction [63].

### 7.3. Neuroprotection

**Figure 5:**
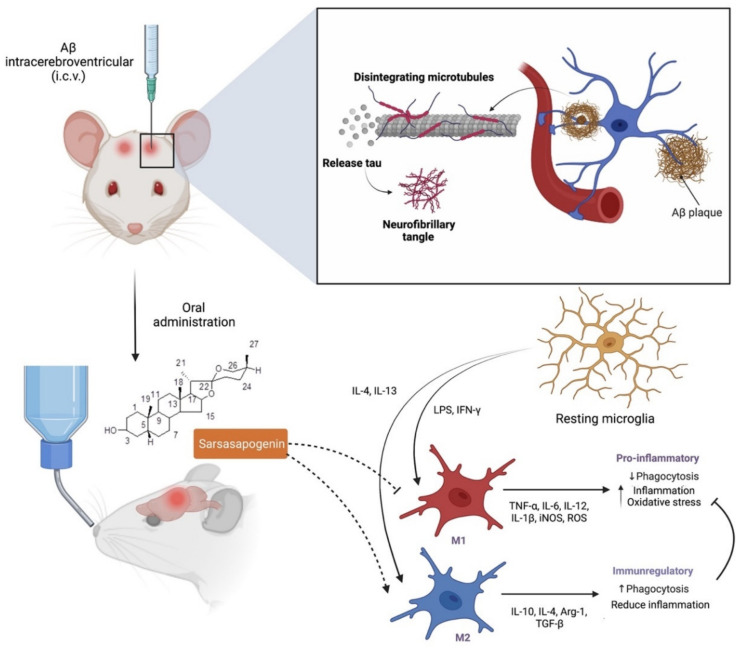
Neuroprotection effect of sarsasapogenin. Sarsasapogenin may suppress the expression of pro-inflammatory M1 markers while elevating the expression of anti-inflammatory M2 biomarkers when taken orally. M2 promotes to the additional inhibition of the M1 marker, hence, assisting in the protection of neuroglial cells. IFN-γ, interferon gamma; iNOS, nitric oxide synthase; ROS, reactive oxygen species; Arg-1, arginase 1; TGF-β, transforming growth factor beta.

Alzheimer’s disease (AD), also known as an irreversible neurodegenerative disorder, is specified by the deposition of beta-amyloid (Aβ) in the extracellular as well as phosphorylation of tau protein to the intracellular cells that are associated with the neuronal and synaptic loss, leading to cognitive deterioration and memory loss. The disparity between Aβ production and clearance results in amassment and deposition in the brain that lead to a sporadic AD. Huang et al. [13] demonstrated the (1) therapeutic effect of sarsasapogenin-13, a sarsasapogenin derivative on learning and memory impairments in Aβ-injected mice; (2) the role of sarsasapogenin-13 performed in neuroglia-mediated anti-inflammation and Aβ clearance. Their findings indicated that oral administration of sarsasapogenin reduced memory deficits in mice injected with Aβ intracerebroventricular (i.c.v.) while protecting neuroglial cells against Aβ-induced cytotoxicity. Further mechanical studies revealed that sarsasapogenin inhibits the elevation of pro-inflammatory M1 markers while increasing the expression of anti-inflammatory M2 markers in Aβ-treated cells (Figure 5). Moreover, Aβ clearance through Aβ phagocytosis and breakdown were further aided by sarsasapogenin. Fatty acid translocase (CD36), insulin degrading enzyme (IDE), neprilysin (NEP) and endothelin-converting enzyme (ECE) expression in neuroglia were also influenced by sarsasapogenin. It was concluded that the neuroprotective action of sarsasapogenin is related to its modulatory effects on the microglia activation state, phagocytic capabilities and the production of Aβ-degrading enzymes, making it a viable therapeutic agent in the early stages of Alzheimer’s disease [13].

To develop a therapeutic strategy for Alzheimer’s disease, a multi-target directed ligand (MTDL) approach is often necessary. Kashyap et al. [32] selected the aqueous extract of *Asparagus racemosus*, including its secondary metabolite sarsasapogenin, for his investigation, since it has a wide range of therapeutic properties. The team conducted several tests, such as Aβ-induced neurotoxicity on PC12 cells, to confirm its nootropic effect that has been referenced in ancient Ayurvedic literature. They found that sarsasapogenin inhibited key enzymes involved in Alzheimer’s disease pathogenesis, including acetylcholinesterase (AChE), butyrylcholinesterase (BuChE), beta-secretase 1 precursor (BACE1), and monoaminoxidase-B (MAO-B) in a concentration-dependent way [32].

In vitro research also revealed that sarsasapogenin has anti-amyloidogenic, anti-oxidant, and neuroprotective properties. For AChE and BuChE, the IC_50_ values of sarsasapogenin were 7.7 and 23.4 μM, respectively. Sarsasapogenin markedly prevented peptide nucleation and fibril formation as confirmed by transmission electron microscopy (TEM) imaging of Aβ aggregates as short and scattered fibrils. Furthermore, sarsasapogenin protects PC12 cells from beta amyloid 1-42 (βA42) and hydrogen peroxide (H_2_O_2_)-mediated cytotoxicities. Likewise, MAO-B and BACE1 enzymes were inhibited by sarsasapogenin in a concentration-dependent manner. Based on molecular docking studies, sarsasapogenin significantly binds to the catalytic sites of numerous targets (AChE, BuChE, Aβ42, BACE1, and MAO-B), suggesting that it has disease-modifying effects against Alzheimer’s [32].

In another study, Zhang et al. [64] reported that sarsasapogenin ameliorates learning and memory and the loss of neurons as well as reduces neurogenesis in the hippocampus. The said findings were confirmed in streptozotocin-induced type-1 diabetic rats. High glucose-cultured SH-SY5Y cells were exposed to sarsasapogenin (20 and 60 mg/kg) for 9 weeks. Sarsasapogenin was found to enhance learning and memory and reduce neurogenesis as well as neuronal loss in the hippocampus. Sarsasapogenin also inhibited the Aβ overproduction in the hippocampus and cerebral cortex of diabetic rats and in the high glucose-cultured SH-SY5Y cells. The said effect was attributed to the (1) upregulation of BACE1 in protein and mRNA (2) tau hyperphosphorylation from inactivation of the protein kinase beta/glycogen synthase kinase-3β (AKT/GSK-3β) cascade and (3) peroxisome proliferator-activated receptor gamma (PPARγ) antagonism that abolished sarsasapogenin’s effects on key molecules. Furthermore, it was discovered that high glucose-stimulated Aβ overproduction occurred prior to tau hyperphosphorylation in neurons. Hu et al. [65] further confirmed that sarsasapogenin can enhance learning and memory. Interestingly, sarsasapogenin neither hindered acetylcholinesterase nor occupied the binding sites of the muscarinic acetylcholine receptor (M receptor), indicating that it is neither a cholinesterase inhibitor nor an agonist or antagonist of M receptors. The densities of total M receptor and its M1 subtype in the brain of the three models, however, were considerably lower than that shown by the control rats. Sarsasapogenin markedly increased the densities of total M receptor and its M1 subtype. The investigation done by an autoradiography using 3H-pirenzipine further confirmed that the density of M1 subtype located in the cortex, hippocampus as well as striatum in elderly rats were remarkably lowered, yet can be reversed to normalcy by sarsasapogenin’s administration.

Interactions between molecular and cellular abnormalities, as well as genetic and environmental variables, may promote the formation of depression and stress-related mental disorders [66]. However, Islam et al. [67] found that taking an antidepressant increases the activation of nicotinic acetylcholine receptors (nAChRs), which serve as excitatory cation channels and include α-subunits (α2–α7) and β-subunits (β2–β4) [68,69]. The 4 and α7 subunits of nAChRs expressed in the brain are the most noticeable. The α4-nAChR receptor can be found all across the nervous system, but it is concentrated in the hippocampus, cerebral cortex, and several brainstem nuclei [70,71]. Nicotine or agonists of the 7- and 4-nAChRs, according to a mouse study, may enhance the antidepressant’s beneficial effects. Sarsasapogenin enhances memory in a memory-impaired rat model by increasing the density of acetylcholine receptors in the brain. The behavioural despair test confirmed that sarsasapogenin had antidepressant-like effects in mice [25]. The antidepressant effect of sarsasapogenin and its relation to cholinergic signalling were investigated by Feng et al. [14]. The olfactory bulbectomized (OB)-induced sucrose preference deficit was significantly recovered by sarsasapogenin (20 and 40 mg/kg) and amitriptyline. In comparison to the Sham group, OB rats displayed less grooming and more evident hyperactivity, as measured by increased ambulation and rearing. The OB-induced enhanced locomotor activity was considerably reduced by sarsasapogenin (20 and 40 mg/kg) and amitriptyline (10 mg/kg) therapy. Administration of sarsasapogenin (20 and 40 mg/kg) significantly enhanced 7- and 4-nAChRs protein levels in OB rats. Hence, sarsasapogenin can restore depression-like behaviours caused by OB and improve cholinergic system malfunction. It is plausible that the antidepressant activity of sarsasapogenin is mediated by an increase expression in 7- and 4-nAChRs, as well as by affecting AChE activity. Overall, the data indicate that cholinergic signalling is a promising target for the development of a sarsasapogenin therapy for depression.

Ren et al. [25] reported that sarsasapogenin reduced the duration of immobility in a dose-dependent manner in a forced swim test. Nevertheless, since sarsasapogenin did not generate hyperlocomotion in the open-field test, its antidepressant effect cannot be attributed to an increase in motor activity. On the other hand, sarsasapogenin increase the concentration of 5-HT (serotonin) in the brain, conversely reducing its turnover possibly due to monoamine metabolism. Noradrenaline levels tend to increase in the hypothalamus, following the treatment with sarsasapogenin (25 and 50 mg/kg) indicating that sarsasapogenin has an antidepressant effect by regulating the 5-HT and noradrenaline systems. The activity of MAO-A influenced the release of neurotransmitter such as 5-HT and noradrenaline that are crucial in ameliorating depression. Further, Zhang et al. [72] explored the effects and the possible mechanisms of sarsasapogenin in protecting high glucose-induced amyloid-beta (Aβ) peptide overproduction in HT-22 cells where there was increased Aβ expression and Aβ42 levels, as well as elevated BACE1 protein, mRNA levels and enzymatic activities which were ameliorated following sarsasapogenin administration. Administration of moderate and high doses of sarsasapogenin (5 and 25 mol/L) reduced PPARγ levels that were increased in the presence of glucose. Sarsasapogenin also possesses substantial neuroprotective effects as confirmed by their study in which it (1) inhibits Aβ deposit and (2) increase cell survival in high glucose cultured HT-22 cells, likely to be mediated by PPAR activation and to be associated with BACE1 downregulation.

The effect of sarsasapogenin (6 mg/kg) on nootropic and neutrophic as well as their mechanisms, in vitro as well in vivo was investigated by Dong et al. [12]. For the former, MTT assays were used to determine the viability of rat primary astrocytes treated with sarsasapogenin and neurons cultured with conditioned medium of sarsasapogenin-treated rat primary astrocytes. As for the latter, scopolamine-induced cognitive deficit model was used where the mice’s spatial memory was tested using a Morris water maze. Based on their findings, sarsasapogenin promoted the viability of primary astrocytes and sarsasapogenin-treated astrocyte within the conditioned medium and improved the survival rate of primary neurons. Remarkably, sarsasapogenin significantly increased BDNF levels in astrocytes. Sarsasapogenin also alleviated cognitive impairments in animal models and elevated BDNF and PSD95 levels in the brain.

Overall, sarsasapogenin modulates growth factors, enzymes, transcription factors, kinase, inflammatory cytokines, and proapoptotic (by upregulation) and antiapoptotic (by downregulation) proteins. Sarsasapogenin alone or in combination with other drugs, could be a promising treatment for various types of cancer.

### 7.4. Antidiabetic Activity

**Figure 6:**
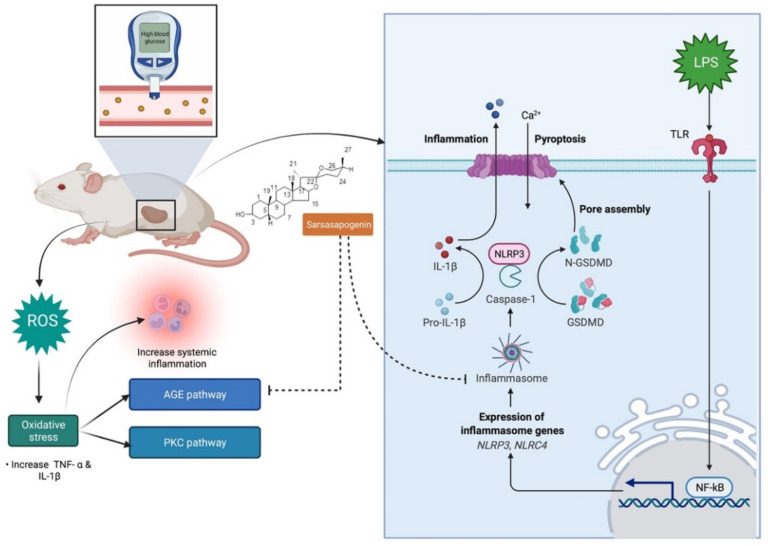
Sarsasapogenin mechanism of action in inflammasome activation and AGE. NLRP3 is triggered by a wide variety of stimuli, including LPS via Toll-like receptors (TLR), which results in the manufacture of the cytokine precursor via NF-kB and other inflammasome constituents, including NLRP3. Sarsasapogenin inhibits the activation of the NLRP3 inflammasome and the AGE pathway, vastly improving diabetic nephropathy in rats. Abbreviations: N-GSDMD, N-terminal Gasdermin-D; GSDMD, Gasdermin-D; NLRP3, NLR family pyrin domain containing 3; NLRC4, NLR Family CARD Domain Containing 4; AGE, Advanced glycation end products; PKC, Protein kinase C.

Diabetic nephropathy (DN) is a chronic complication of diabetes mellitus (DM) and is the main cause of end-stage renal disease [73]. The pathological effects of DN is related with increased nucleotide binding and oligomerisation domain-like receptor family pyrin-containing protein 3 (NLRP3) inflammasome [74]. The rise in the levels of inflammatory mediators is associated with an elevation of coagulability and the drift towards the formation of thrombus in patients diagnosed with type 2 DM who have microvascular complications [75]. Tang et al. [30] in their recent study, investigated the effects of thrombin and/or its receptor, protease-activated receptor 1 (PAR-1) on both NLRP3 inflammasome and NF-κB signalling in DN patients, as well as their molecular mechanism. Sarsasapogenin (0, 20 and 60 mg/kg, p.o. for 10 weeks) substantially reduced protease-activated receptor (PAR-1) protein and mRNA levels in the kidney but had no effect on thrombin activity, despite the fact that thrombin activity was significantly reduced in the renal cortex of diabetic rats. Further, the protective effects of sarsasapogenin against early stage DN in rats [26]. Prolonged treatment with sarsasapogenin (0, 20 and 60 mg/kg, p.o. for 9 weeks) dramatically improved the renal dysfunction, as evidenced by the lower albuminuria, kidney weight index, serum uric acid and morphologic alterations such extracellular matrix growth and accumulation (fibronectin and collagen IV levels). There was also lower levels of interleukin 18, NLRP3, activated caspase 1, as well as advanced glycation end products (AGEs) and their receptor (RAGE) in the renal cortex which were not seen in control rats. Overall, the researchers concluded that sarsasapogenin inhibits NLRP3 inflammasome activation and the AGEs–RAGE interaction, which can significantly improve DN in rats [26] (Figure 6). Li et al. [2] further explored the action of sarsasapogenin in restoring podocyte autophagy in DN by targeting glycogen synthase kinase 3 beta pathway (GSK3β) signalling pathway. Podocytes are cells involved in glomerular filtration, and therefore podocytes injury contributes to proteinuric kidney disease that is related with the development of ND. Sarsasapogenin (1) ameliorates podocyte injury and autophagy in the presence of high glucose concentration in DN and (2) plays a vital role in GSK3β signalling pathways. Therefore, it is a potential agent for the DN therapy. Since podocyte injury caused by an aberrant podocyte autophagy is an important process in DN, restoring podocyte autophagy is a viable method for treating DN. Therefore, the effects of sarsasapogenin on podocyte injury in diabetic rats, as well as the mechanisms involved were further investigated in mouse podocytes treated with high glucose (40 μM). Sarsasapogenin (60 mg/kg, p.o. for 10 weeks) ameliorated autophagy-related proteins including ATG5, Beclin1 and LC3B as well as podocyte marker proteins such as podocin, nephrin and synaptopodin in the diabetic kidney. Subsequently, a network pharmacology used to evaluate GSK3 as a potential target for sarsasapogenin’s impact on DN, confirmed the significant changes in GSK3 signalling seen in the diabetic kidneys.

The effects of sarsasapogenin on damaged vascular endothelium in high-glucose cultured human umbilical vein cultured cells (HUVECs) related to the gestational diabetes mellitus (GDM) were explored by Liu et al. [76]. GDM is a disease of uncommon glucose tolerance during pregnancy which may lead to fetal macrosomia, respiratory distress syndrome as well as type 2 diabetes in the offspring. The pathogenic alterations of these pathways were investigated using the umbilical cord and plasma of GDM patients, as well as high glucose HUVECs. Meanwhile, HUVECs were used to explore the effects and the possible mechanism of action of sarsasapogenin. Interestingly, co-treatment with sarsasapogenin affect the thrombin/PAR-1 pathway, AGEs/RAGE axis and reduced the NLRP1 inflammasome. Endothelial damage in GDM was likely to be caused by the increased interaction between the AGEs/RAGE axis and the thrombin/PAR-1 pathway, followed by activation of the NLRP1 inflammasome. Sarsasapogenin is also protective against endothelial injury in patients with chronically high glucose level. Through down-regulation of the PAR-1 receptor, Kong et al. [77] studied the effects of sarsasapogenin on diabetes-associated memory impairment and neuroinflammation. In streptozotocin-induced diabetic rats, sarsasapogenin (20 and 60 mg/kg, p.o. for 8 weeks) alleviated diabetes-related memory impairment, as evidenced by higher platform crossings and percentage of time spent in the target quadrant in Morris water maze tests. In the hippocampus and cerebral cortex, sarsasapogenin blocked the nucleotide-binding domain and leucine-rich repeat-containing protein 1 (NLRP1) inflammasome, repressed the AGEs/RAGE axis, and decreased the upregulation of the thrombin receptor PAR-1. In high glucose cultured SH-SY5Y cells, sarsasapogenin also reduced high hyperglycemia-induced neuronal damage, activation of the NLRP1 inflammasome, and PAR-1 upregulation, but it had no effect on thrombin activity. Thus, sarsasapogenin was found to alleviate diabetes-induced memory impairment by reducing neuroinflammation caused by the activated NLRP1 inflammasome and NF-κB, which is controlled by cerebral PAR-1.

Overall, the preclinical investigations showed that sarsasapogenin can enhance glucose homeostasis and minimise the diabetes phenotype by lowering blood glucose levels and decreasing insulin resistance. However, more research is needed to completely comprehend the effects of sarsasapogenin in specific body tissues such as skeletal muscle, adipose tissue, liver, and pancreatic β-cells. Further, sarsasapogenin appears to be a promising candidate for clinical use in the treatment of insulin resistance and type 2 diabetes. Hence, clinical investigations on sarsasapogenin are also necessary.

### 7.5. Anti-Osteoclastogenic Activity

Both osteoclasts and osteoblasts govern bone homeostasis [78]. Osteoclasts are polynuclear cells that are responsible for bone resorption. An increased number of osteoclasts and rapid bone resorption influence osteoclast-related bone-lytic illnesses including osteoporosis. Osteoclasts produce tartrate-resistant phosphatase (TRAP) that adheres to the bone surface via an actin-binding sealing zone, releasing proteases, such as tissue protease K, and causing the mineralised bone matrix to degrade. The two cytokines that regulate osteoclast differentiation are macrophage-colony-stimulating factor (M-CSF), which (1) is required for cell proliferation and survival in monocyte cell lines, (2) enhances bone marrow precursor development, and (3) boosts receptor activator of nuclear factor-B ligand (RANKL) expression in bone marrow cells, and RANKL which is the primary cytokine required to promote monocyte fusion that is essential in the formation of multinucleated osteoclasts [79,80,81,82]. Osteoclastogenesis occurs when the RANK receptor is activated by its ligand RANK, where the interaction initiates a signal cascade that results in the expression of nuclear factor of activated T cells 1 (NFATc1), a key regulator for osteoclastogenesis. NFATc1 also activates the pathways of PI3K/Akt, MAPK/AP-1, and NF-B [83,84,85]. Peng et al. [23] conducted a study to determine the effect of sarsasapogenin on the inhibition of osteoclastogenesis as well as the prevention on the bone loss through the NF-κB B and JNK/MAPK signalling pathways. Sarsasapogenin (5 and 10 mg/kg, s.c.) suppressed the activation of the major osteoclast transcription factor NFATc1 by blocking several RANKL-induced signalling cascades. The in vitro and in vivo findings in the mouse osteolysis model were consistent, where sarsasapogenin confers protection against the bone loss contributed by LPS. Overall, the findings support the use of sarsasapogenin as a novel therapy for osteoclast-related osteolytic disorders.

Based on the information shown above, sarsasapogenin, which has anti-inflammatory and immunomodulatory effects, could be used to treat osteoclastic disorders. However, more research is needed, particularly on its mechanism of action and pharmacokinetics, in order to validate its safety and efficacy and then to move forward with drug development.

### 7.6. Treatment for Precocious Puberty

Precocious puberty is the premature activation of gonadotropin-releasing hormone (GnRH) affecting growth and development [51]. It is also described as the manifestation of secondary sex traits in girls before the age of eight or in boys before the age of nine [86]. Due to the early beginning of puberty, early growth and development as well as reduced bone growth years, children with early puberty tend to be small in stature. Additionally, the disease can lead to a variety of psychological and physical issues in patients and has been linked to metabolic disorders such as diabetes, cardiovascular disease as well as breast and prostate cancers [87,88,89].

Currently, gonadotropin-releasing hormone analogue (GnRHa) is commonly utilised to treat prematured puberty [90] with leuprolide most often used against precocious puberty. Leuprolide (1) acts on the pituitary in a constant non-pulsed manner, (2) downregulates the GnRH receptor, (3) limits the sensitivity to the GnRH receptor, and (4) inhibits the pituitary to secrete luteinizing hormone (LH) and follicle-stimulating hormone (FSH), overall slowing secondary sex development [91,92]. However, when taken for the first time, it may cause vaginal bleeding with an incidence of 16–60% [93] and increases the risk of polycystic ovary syndrome [52]. Therefore, the role of alternative therapies is important. Hu et al. [27] investigated the effects of sarsasapogenin in treating precocious puberty by regulating the hypothalamus–pituitary–gonadal (HPG) axis. To establish the precocious puberty phase, 5 day old rats were given a single subcutaneous injection of danazol (300 µg). After 10 days, sarsasapogenin (4 mg/kg) was administered via oral gavaging for 10 days. Danazol affects the reproductive system of the rat and can rapidly stimulate the activation of the HPG axis to promote precocious puberty. However, sarsasapogenin prevents the development of gonads and downregulates the expression of GnRH and gonadotropin-releasing hormone receptor (GnRH-R) via the Kiss-1/GPR54 pathway, hence, delaying the activation of the HPG axis and exerting its therapeutic effects.

Payment endocrinologists face a difficult task in assessing and managing this disease [94]. The Pediatric Endocrinology Society has suggested that the combination of GnRHa and recombinant growth hormone (rhGH) should not be recommended as a routine treatment due to the fact of its high cost and the lack of large-scale randomised clinical trials evaluating the safety and effectiveness of this combination therapy [95]. Based on the above studies, sarsasapogenin is a potential molecule to alleviate precocious puberty. To support its therapeutic application, more information about the potential mechanism of action of sarsasapogenin is needed.

## 8. Bioavailability and Pharmacokinetics of Sarsasapogenin

The pharmacokinetics of sarsasapogenin were evaluated in rats after intragastric injection of 25, 50, and 100 mg/kg [96]. This study measured sarsasapogenin plasma concentrations for 72 h. The results obtained were compared to the major steroidal saponins extracted from *A. asphodeloides*, such as timosaponin H1, timosaponin E1, timosaponin E, timosaponin B-II, timosaponin B-III and anemarrhenasaponin I, based on the study from Liu et al. 2015 [97]. After taking the sarsasapogenin, the sarsasapogenin content in the blood slowly increased. As consequence, the time for maximum plasma concentration (T_max_) of sarsasapogenin was significantly longer than the primary steroidal saponins in *A. asphodeloides* (3.17–4.76 h). Sarsasapogenin has a low aqueous solubility, and this is one of the main reasons for its poor absorption rate. Regarding the plasma half-life (t_1/2_), a single intragastric 100 mg/kg sarsasapogenin dosage in rats resulted in a t_1/2_ of 17.72 h compared to the major steroidal saponins (15.1–16.1 h). The results indicating that sarsasapogenin may have an incredibly long residence time shows that sarsasapogenin has low bioavailability; thus, the excretion of sarsasapogenin is delayed. The linearity of the area under the plasma concentration versus time curve (AUC_0–72h_) and maximum plasma concentration (C_max_) to dosages performed well in regression analysis. As doses increased, the parameters of t_1/2_ and t_max_ were not remarkably affected. A dose range of 25–100 mg/kg of sarsasapogenin was shown to be linear in pharmacokinetics [97].

## 9. Metabolism of Sarsasapogenin

Sarsasapogenin and smilagenin were hydrolysed in the rumen to yield the parent sapogenins, which were then oxidised and reduced at C-3 to provide the equivalent epi-sapogenins. Because of the reduced amount of sapogenins found in the small intestine and the identification of significant amounts of sapogenins, such as smilagenone, sarsasapogenin, smilagenin, episarsasapogenin and smilagenone, in the liver, this shows that these sapogenins were absorbed from the upper intestine and transported to the liver via the portal vein, where smilagenin and sarsasapogenin that had not been oxidised and reduced in the rumen were oxidised and reduced, and this activity is referred to as phase 1 metabolism, before *epi*-analogues were conjugated and excreted into the bile as episarsasapogenin and epismilagenin (phase II metabolism) [37].

## 10. Sarsasapogenin-Derived Compounds

**Figure 7:**
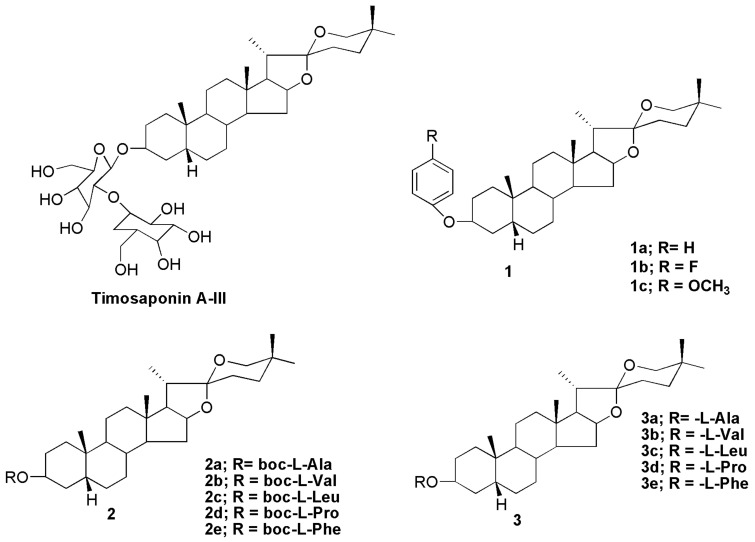
Chemical structures of sarsasapogenin-derived compounds (**2a**–**2e** and **3a**–**3e**).

Steroidal saponins are found in a wide range of plant species. Their different structures have led to a wide range of applications including the manufacture of new drugs. The non-saccharide and oligosaccharide sections of the saponin molecule have been suggested to confer important characteristics of individual saponins. For example, saponins of the spirostane type are monodesmosidic glycosides with six rings (i.e., A–F) that are similar to sapogenin. They are distinguishable by an axially oriented methyl or hydroxymethyl (C-27) on the F ring. Spirostane glycoside sapogenins have a cis- or trans- fusion between rings A and B, or a double bond between C-5 and -6, resulting in different subtypes such as 5α (neotigogenin), 5β (sarsasapogenin) and D5 (narthogenin). A galactose–glucose disaccharide molecule was linked to the C3 position of the aglycone sarsasapogenin in timosaponin A-III (TAIII), a spirostanol saponin. Timosaponin A-III is a promising bioactive lead molecule isolated from the rhizome of *Anemarrhena asphodeloides* for cancer therapy. Structural alteration at the C3 and C26 positions of sarsasapogenin has traditionally been the main focus for structure–activity investigations [16]. Etherification of the C3 hydroxyl, with appropriate benzyl bromides, yielded compounds **1a**, **1b** and **1c** (Figure 7) that can be employed as intermediates in the manufacture of novel sarsasapogenin derivatives that induce cytotoxicity and apoptosis in human breast cancer cells MCF-7 [16]. The cytotoxicity of tert-butoxycarbonyl (Boc)-protected amino acid esters **2a–2e** and de-protected amino acid esters **3a**–**3e** of sarsasapogenin derivatives was investigated in HepG2 and MCF-7 cells. Only compound **3d** with an L-prolyloxy substituent was cytotoxic to HepG2 and MCF-7 cells, with IC_50_ values of 16.02 and 13.76 µM, respectively [98].

**Figure 8:**
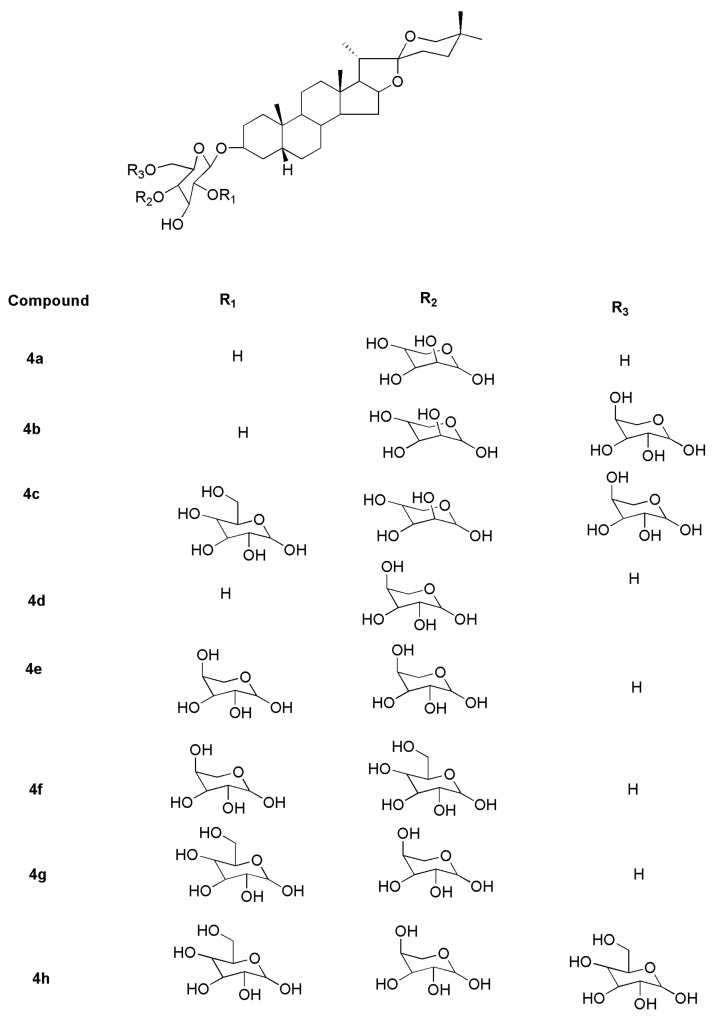
Sarsasapogenin-derived compounds isolated and identified from *Asparagus adscendens* and *Asparagus racemosus*.

Compounds **4a**–**4h** (Figure 8) were isolated and identified from *Asparagus adscendens* and *Asparagus racemosus*. Compound **4a** was identified as 25S-5β-spirostan-3β-yl-*O*-[*O*-β-d-arabinopyranosyl (1→4)]-β-d-glucopyranoside. Compounds **4b** and **4c** were elucidated using spectral techniques and were determined to be spirostanol glycosides asparanins C and D. The glycosides **4d** and **4e** were identified as 25S-5βspirostan-3β-yl-*O*-[*O*-α-l-rhamnopyranosyl (1→4)]-β-d-glucopyranoside and 25S-5β-spirostan-3β-yl-*O*α-L-rhamnopyranosyl (1→2)-*O*-[*O*-α-l-rhamnopyranosyl-(1→4)]-β-d-glucopyranoside, respectively. Compounds **4f** and **4g** were identified as shatavarin IV and 25S-5β-spirostan-3β-yl-*O*-βd-glucopyranosyl (1→2)-*O*-[*O*-α-l-rhamnopyranosyl-(1→4)]-β-d-glucopyranoside, respectively. Compound **4h** was reported as 25S-5β-spirostan-3βyl-*O*-β-d-glucopyranosyl (1→2)-*O*-{[*O*-β-d-glucopyranosyl-(1→4)]-*O*-[α-d-arabinopyranosyl (1→6)]}-β-d-glucopyranoside [46].

**Figure 9:**
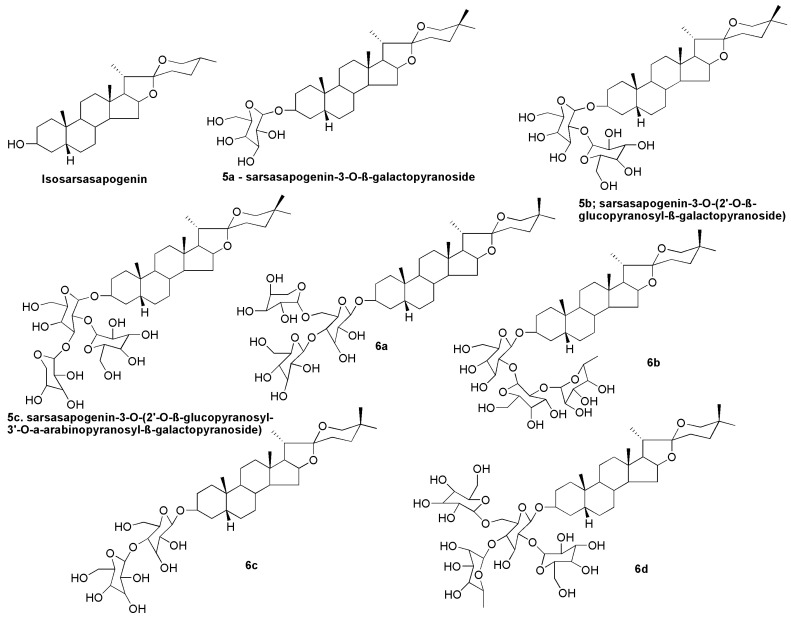
Chemical structures of isosarsasapogenin and other sarsasapogenin-derived compounds (**5a**–**5c** and **6a**–**6d**).

The stereoisomer of sarsasapogenin, also known as isosarsasapogenin, was synthesised to investigate the in vivo metabolism profile [45]. The individual pure saponins isolated from the flowering tops of *Narthecium ossifragum* [99] were identified and reported as sarsasapogenin-3-*O*-β-galactopyranoside (**5a**), sarsasapogenin3-*O*-(2′-*O*-β-glucopyranosyl-β-galactopyranoside) (**5b**) and sarsasapogenin-3-*O*-(2′-*O*-β-glucopyranosyl-3′-*O*-α-arabinopyranosyl-β-galactopyranoside) (**5c**) followed by evaluation of their cytotoxic activities. The saponins exhibited cytotoxicity in the micromolar range, and cytotoxicity proportionally increased with the number of glycosyl substituents. Sarsasapogenin glycosides sarsasapogenin-3-*O*-β-d-glucopyranosyl-(1 → 4)-[α-l-arabinopyranosyl- (1 → 6)]-β-d-glucopyranoside (**6a**), (25S)-5β-Spirostane-3β-ol-3-*O*-α-l-rhamnopyranosyl-(1 → 2)-β-d-glucopyranosyl-(1 → 2)-β-d-glucopyranoside (**6b**), curillin G (**6c**) and parillin (**6d**) (Figure 9) were also isolated and reported to be present in the root of *Smilax aspera* subsp. *mauritanica* [55] and *Smilax ornata* [100].

## 11. Challenges and Opportunities in Developing Sarsasapogenin as a Novel Drug Molecule

Drug development is the most important translational research undertaking that contributes to human health and well-being. Drug development refers to all activities involved in transforming a drug-like molecule into a final licenced drug product for marketing by the appropriate regulatory authorities. The phases of drug discovery and development that involve identifying and screening small molecules for their therapeutic and biological properties are essential. Most of the world’s well-known compounds have been documented in a library that has been developed over time. The majority of such molecules or small molecules were previously derived from natural resources. Currently, researchers have access to a wide spectrum of chemical substances, and high-throughput and combinatorial chemical procedures have covered thousands of unique molecules. It is yet unknown which of these millions of molecules possess the properties that will enable them to become drugs [4].

The physicochemical features of sarsasapogenin were evaluated by employing Lipinski’s rule of five to establish its drug-likeness appearance. Based on the rule, any drug-like compound should have (1) a molecular weight of ≤500; (2) a partition coefficient (log *p*)-value of ≤5; (3) H-bonds donors of ≤5; (4) H-bonds acceptors of ≤10; (5) rotatable bonds of ≤10. The characteristics are important for improved folding, polarity, or molecular size, and the drug-like molecules are purported to confer some therapeutic benefits. Overall, sarsasapogenin matches all of Lipinski’s five drug-likeness criteria, with the exception of its partition coefficient value which exceeded five (Table 3) due to the fact of its hydrophilic nature. Nevertheless, the findings suggest that sarsasapogenin could be developed into a promising therapeutic agent for a variety of disorders.

Sarsasapogenin is a hydrophilic molecule that is poorly soluble in body fluid, though phosphorylation or acetylation can improve this said property further. However, since solubility has a significant impact on pharmacokinetics, administration of sarsasapogenin in its free form can cause a rapid deterioration and renal clearance, resulting in a short biological half-life in circulation. Sarsasapogenin also has a drawback in the fact that its distribution is mostly uniform and unspecific, which can lead to side effects and poor drug availability in target tissues. Thus, delivery systems that overcome these obstacles and, in particular, enhance the half-life of sarsasapogenin and deliver them to the site of action is ideal.

**Figure 10:**
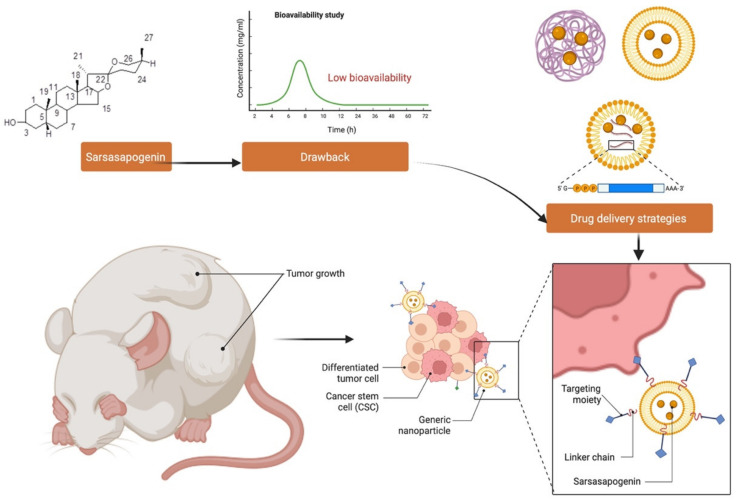
Schematic representation of the drug delivery strategy for sarsasapogenin-loaded liposomes augmented with a targeting moiety, such as cell-penetrating peptides (CPPs), to enhance localisation and bioavailability of the bioactive molecule.

New nanoscale carrier technologies may be able to meet these expectations while also addressing some of the issues associated with sarsasapogenin therapy (Figure 10). However, despite the recent enthusiasm for nanomaterials, depending on the concentration applied, solubility, shape, and size, they may be particularly unsafe, making their utilisation problematic. More effective and focused therapies are possible with the development of novel drug delivery technologies.

By improving drug targeting, pharmacokinetics, efficacy, and cellular uptake, nanomedicine has the ability to bridge the gap between pharmaceutical limits and natural phytochemical therapeutic potentials [9,101,102,103,104,105]. Nanoparticles, such as polylactic-co-glycolic acid (PLGA), polyethylene glycol (PEG), liposomal delivery systems, and RNA-based delivery, provide a number of benefits including (1) better adsorption; (2) targeting with precision; (3) efficient encapsulation; (4) improved bioavailability; (5) fewer side effects; (6) more stability [106,107,108]. The new delivery systems provide a number of possibilities for achieving tissue selectivity and minimising system exposure, which are both necessary for deploying new pharmacological molecules derived from sarsasapogenin for enhanced therapy. Many promising results have been reported in recent years, but clinical translation remains a barrier and progress remain slow. It is important to remember that while delivery vehicles can help minimize sarsasapogenin-related issues, they may also cause other problems due to the materials used. Hence, every nanoformulation should be examined not only for the therapeutic efficacy and minimised drug side effects but also for material-related toxicity and immunological reactions. Since this is a time-consuming process that must pass many stages of preclinical and clinical developments, it is not surprising that not many clinical studies have been conducted especially on natural products. Nevertheless, some findings are largely favourable, leading to anticipation of even more breakthroughs, especially those involving the use of sarsasapogenin.

## 12. Conclusions and Future Perspectives

Overall, sarsasapogenin is a key sapogenin isolated from *Anemarrhena asphodeloides* Bunge, and it could be used to treat a range of health problems with safety and efficacy. The distribution of sarsasapogenin in medicinal plants as well as its isolation, structural characterisation, physicochemical properties and biosynthesis were discussed in this review. In addition, this review also provided scientific evidence on its pharmacology and therapeutic potential. Sarsasapogenin has anti-inflammatory, anticancer, antidiabetic, anti-osteoclastogenic, and neuroprotective properties, and it could be used to treat precocious puberty. However, existing research mostly focused on the pharmacological actions; hence, interpretation of molecular mechanisms of its activities is still insufficient. The exceptional anti-inflammatory capability of sarsasapogenin may mediate many of its therapeutic potentials as evidenced by the described investigations. Sarsasapogenin also has significant cytotoxic and apoptotic capabilities that enhance its anticancer actions. However, more research is needed to fully comprehend the mechanisms of sarsasapogenin in inhibition of cancer cell line proliferation. There is insufficient evidence on the toxicity evaluations of this molecule, necessitating further research in the future to ensure its safety. Furthermore, because many of the existing literature are only in vitro and in vivo, however, more preclinical and clinical research is required to fully comprehend these activities, identify the underlying molecular mechanisms and to see if the same benefits may be seen in humans. Despite several changes and semi-synthetic investigations, additional research is needed to better understand the structure–activity relationship and generate sarsasapogenin derivatives with improved activities. Furthermore, extensive research into the pharmacokinetics and bioavailability of sarsasapogenin is needed to prove its safety and efficacy in the treatment of numerous disorders. We hope that this review may provide some direction for future drug design, development and therapy of sarsasapogenin.

## Figures and Tables

**Table 1:** List of medicinal plants containing sarsasapogenin.

Plant Family	Genus	Plant Species	References
Asparagaceae	*Anemarrhena*	*Anemarrhena asphodeloides*	Wang et al. [11]; Moon et al. [22]; Peng et al. [23]; Pan et al. [24]; Ren et al. [25]; Liu et al. [26]; Hu et al. [27]; Lim et al. [28]; Bao et al. [29]; Tang et al. [30]; Pei et al. [31]
	*Asparagus*	*Asparagus racemosus*	Kashyap et al. [32]
		*Asparagus officinalis*	Paczkowski and Wojciechowski [33]; Huang et al. [34]
		*Asparagus cochinchinensis*	Okanishi et al. [35]; Zhang et al. [36]
	*Yucca*	*Yucca schidigera*	Flåøyen et al. [37]
Smilacaceae	*Smilax*	*Smilax china*	Ingawale et al. [38]
		*Smilax ornata*	Ingawale et al. [38]; Power and Salway [39]; Mandlik et al. [40]
		*Smilax aspera*	Ingawale et al. [38]; Mandlik et al. [40]
		*Smilax febrifuga*	Ingawale et al. [38]; Mandlik et al. [40]
		*Smilax aristolochiifolia*	Ingawale et al. [38]; Mandlik et al. [40]
		*Smilax officinalis*	Ingawale et al. [38]
		*Smilax medica*	Marker et al. [41]
Nartheciaceae	*Narthecium*	*Narthecium ossifragum*	Uhlig et al. [42]; Ceh and Hauge [43]
Ranunculaceae	*Helleborus*	*Helleborus niger*	Duckstein and Stintzing [44]

**Table 2:** ^1^H-NMR and ^13^C-NMR data for sarsasapogenin in deuterated chloroform (CDCl_3_).

Carbon	Signal (δ)	Proton	Signal (δ) (Multiplicity)
1	30.1	1	1.49 (m), 1.24 (m)
2	37.0	2	1.72 (m), 1.47 (m)
3	67.2	3	4.10 (br. s)
4	33.7	4	1.67 (m), 1.41 (m)
5	36.7	5	1.42 (m)
6	26.7	6	2.1 (m), 2.7 (m)
7	26.7	7	1.52 (m), 1.27 (m)
8	35.4	8	1.56 (m)
9	40.0	9	1.40 (d)
10	35.4	10	-
11	21.0	11	1.35 (m)
12	40.5	12	1.14 (m), 1.65 (m)
13	40.8	13	-
14	56.6	14	1.18 (m)
15	31.9	15	1.73 (m), 1.46 (m)
16	81.2	16	4.40 (td, *J* = 7.9, 7.3, 6.2 Hz)
17	62.2	17	1.79 (m)
18	16.2	18	0.75 (s)
19	24.1	19	0.97 (s)
20	42.3	20	1.26 (m)
21	14.5	21	1.07 (d, *J* = 7.1 Hz)
22	109.9	22	-
23	28.0	23	1.95 (m)
24	25.9	24	1.66 (m), 1.44 (m)
25	27.2	25	1.77 (m)
26	65.3	26	3.94 (dd, *J* = 11.0, 2.8 Hz), 3.29 (d, *J* = 10.9 Hz)
27	16.6	27	0.98 (d, *J* = 6.4 Hz)

**Table 3:** Physicochemical and drug-likeness properties of sarsasapogenin.

Property/Rule	Result
Molecular formula	C_27_H_44_O_3_
Molecular weight	416.6 g/mol
Hydrogen bond donors	1
Hydrogen bond acceptors	3
Rotatable bonds	0
Log P (partition coefficient, predicted value)	7.306
Molar refractivity	122.07 cm^3^
Topological polar surface area	38.7 Å^2^

## Data Availability

The data presented in this study are available upon request from the corresponding author.
